# Radiation-induced occult insufficiency fracture or bone metastasis after radiotherapy for cervical cancer? The nomogram based on quantitative apparent diffusion coefficients for discrimination

**DOI:** 10.1186/s40644-020-00353-8

**Published:** 2020-10-23

**Authors:** Xi Zhong, Huali Jiang, Hui Mai, Jialin Xiang, Jiansheng Li, Zhiqing Huang, Songxin Wu, Liangping Luo, Kuiming Jiang

**Affiliations:** 1 Department of Medical Imaging, Affiliated Cancer Hospital & Institute of Guangzhou Medical University, Guangzhou, 510095 China; 2 Department of Cardiovascularology, Tungwah Hospital of Sun Yat-Sen University, Dong cheng East Road, Dong guan, 523110 Guangdong China; 3 Department of Radiology, The Third Affiliated Hospital of Guangzhou Medical University, Guangzhou, 510150 China; 4 Department of Radiology, Guangdong Women and Children Hospital, Guangzhou, 510000 China; 5 Department of Medical Imaging, First Affiliated Hospital of Jinan University, Guangzhou, 510000 China

**Keywords:** Insufficiency fracture, Radiotherapy, Apparent diffusion coefficient, Nomogram, Cervical cancer

## Abstract

**Background:**

Radiation-induced insufficiency fractures (IF) is frequently occult without fracture line, which may be mistaken as metastasis. Quantitative apparent diffusion coefficient (ADC) shows potential value for characterization of benign and malignant bone marrow diseases. The purpose of this study was to develop a nomogram based on multi-parametric ADCs in the differntiation of occult IF from bone metastasis after radiotherapy (RT) for cervical cancer.

**Methods:**

This study included forty-seven patients with cervical cancer that showed emerging new bone lesions in RT field during the follow-up. Multi-parametric quantitative ADC values were measured for each lesion by manually setting region of interests (ROIs) on ADC maps, and the ROIs were copied to adjacent normal muscle and bone marrow. Six parameters were calculated, including ADC_mean_, ADC_min_, ADC_max_, ADC_std_, ADC_mean_ ratio (lesion/normal bone) and ADC_mean_ ratio (lesion/muscle). For univariate analysis, receiver operating characteristic curve (ROC) analysis was performed to assess the performance. For combined diagnosis, a nomogram model was developed by using a multivariate logistic regression analysis.

**Results:**

A total of 75 bone lesions were identified, including 48 occult IFs and 27 bone metastases. There were significant differences in the six ADC parameters between occult IFs and bone metastases (*p* < 0.05), the ADC ratio (lesion/ muscle) showed an optimal diagnostic efficacy, with an area under ROC (AUC) of 0.887, the sensitivity of 95.8%, the specificity of 81.5%, respectively. Regarding combined diagnosis, ADC_std_ and ADC_mean_ ratio (lesion/muscle) were identified as independent factors and were selected to generate a nomogram model. The nomogram model showed a better performance, yielded an AUC of 0.92, the sensitivity of 91.7%, the specificity of 96.3%, positive predictive value (PPV) of 97.8% and negative predictive value (NPV) of 86.7%, respectively.

**Conclusions:**

Multi-parametric ADC values demonstrate potential value for differentiating occult IFs from bone metastasis, a nomogram based on the combination of ADC_std_ and ADC_mean_ ratio (lesion/muscle) may provide an improved classification performance.

## Background

Cervical cancer is one of the most common gynecologic malignant tumors worldwide. In china, the incidence of cervical cancer is ranked as No.1 among gynecological cancers, with approximately 100,000 new cases and 30,500 deaths annually, respectively [1]. Radiotherapy (RT) has been proven as an effective method for the treatment of cervical cancer, and with the introduction of concurrent radiochemotherapy, the patient’s survival has been significantly improved. Meanwhile, the late adverse events associated with RT has drawn more attention, including RT-induced insufficiency fracture [2]. RT-induced IF is a relatively common complication for cervical cancer, previous studies showed that the 2-year cumulative incidences of IF after pelvic RT ranged from 14 to 36.9% [3–7].

Diagnosing postradiation IF accurately and correctly differentiating IF from bone metastasis are particularly important in clinical practice. MRI is a sensitive technique for the assessment of the soft-tissue mass and its associated bone marrow infiltration [8]. Recently, MRI plays a key role in the detection of IF. Compared with bone scintigraphy or computed tomography, MRI demonstrated a preferable diagnostic sensitivity and specificity [9–11]. MRI has limitation in the detection of fracture line, and abnormalities on MRI may sometimes be mistaken for metastatic diseases [5, 11, 12]. Thus, as for the diagnosis of occult IFs (without fracture line), it may be still challenging by using conventional MRI alone.

Diffusion-weighted Imaging (DWI) is an imaging technique that probes the characteristics of biological tissues based on the diffusion properties of water molecules. Several studies recommended DWI as a valid technique in discrimination of benign and malignant marrow lesions [13–15]. However, only qualitative DWI assessment could not exclude the possibility that the T2 shine-through effect might have influence on the appearance of such images. Considering this limit, some studies have quantified the diffusion of marrow lesions by using the apparent diffusion coefficient (ADC) value, and the results indicated that ADC measurement was a reliable method in differentiating benign from malignant vertebral compression fractures [16, 17].

Regarding the differentiation of IF and bone metastasis by using quantitative ADC, a previous study demonstrated that RT-induced IFs showed relatively higher mean ADC value than bone metastases [18]. In addition, our previous study indicated that the mean ADC value showed potential value in the differentiation of IF from bone metastasis, although the diagnostic specificity was limited [19]. However, the value of other ADC parameters (such as ADCmin, ADCmax, ADCstd, relative ADC [lesion to normal bone, lesion to muscle) in the differentiation of IF and metastasis are unclear. Therefore, we aimed to develop a nomogram based on multiparameter ADC values to differentiating occult IF from bone metastasis after RT in cervical cancer.

## Materials and methods

### Patients

**Fig. 1:**
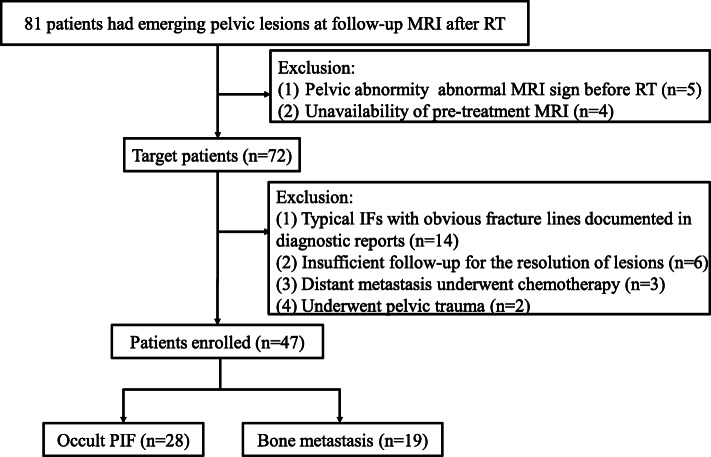
Flowchart of the study population

Institutional review board of Affiliated Cancer Hospital & Institute of Guangzhou Medical University approved this retrospective study, and the patients’ informed consent was waived. We retrospectively reviewed clinical and follow-up MRI data from 856 consecutive cervical cancer patients after RT between January 2011 and December 2017. A total of 81 patients that showed emerging pelvic lesions in RT regions at follow-up MRI were included for further screening. However, 34 patients were excluded according to the following exclusion criteria: (1) unavailability of pre-treatment MRI (*n* = 5) or showing abnormal signal changes in pelvis on pre-treatment MRI (*n* = 4); (2) typical IFs with obvious fracture lines documented in diagnostic reports (*n* = 14); (3) lack of sufficient imaging (MRI and/or CT) follow-up to confirm the nature of the detected lesions (*n* = 6); (4) having received necessitated systemic chemotherapy due to distant metastasis far from the pelvis (*n* = 3), considering that the chemotherapy may alter the signal features of pelvic lesions; (5) had known history of pelvic trauma after RT (*n* = 2). As mentioned in previous studies [11, 19, 20], the reference standard of IF and bone metastasis was based on the combination of all available radiologic findings (MRI and/or CT), clinical data and follow-up for at least 12 months. Biopsy of a bone lesion showed a high risk of fracture or hemorrhage, and the diagnostic performance seemed to be low (14). Finally, a total of 47 patients (female; mean age 57.5 ± 8.4 years) were enrolled in this retrospective study. The detail inclusion flowchart is showed in Fig. 1.

### Magnetic resonance imaging

MRI examinations of the pelvis were acquired in a 1.5 T superconducting magnetic scanner (Intera Achieva; Philips Healthcare). The conventional MRI pulse sequences included axial turbo spin echo (TSE) T1-weighted, axial fat-saturated (FS) TSE T2-weighted, axial and sagittal FS contrast-enhanced T1-weighted. Axial FS DWI was done using a single-shot echo planar imaging with the following parameters: b values of 0 and 800 s/mm^2^; TR/TE 2000/56.1 ms, FOV 32 cm, slice thickness 8 mm, gap 1 mm, and matrix size 256 × 185.

### Quantitative ADC value measurement

DW images were loaded into a post-processing workstation (Extended MR WorkSpace 2.6.3.4; Philips Healthcare, the Netherlands), and the ADC maps were created automatically based on the following formula: ADC = (lnSI0 − lnSI) / (b − b0), where SI0 and SI represents the signal intensity obtained at b0 = 0 s/mm^2^ and b = 800 s/mm^2^, respectively. Two radiologists (J.S.L. with 15 years of experience; H.M. with 10 years of experience) independently measured ADC values in each lesion, adjacent normal bone marrow and muscle. The radiologists were blinded to the clinical data, other imaging findings and the final diagnosis of the lesions.

**Fig. 2:**
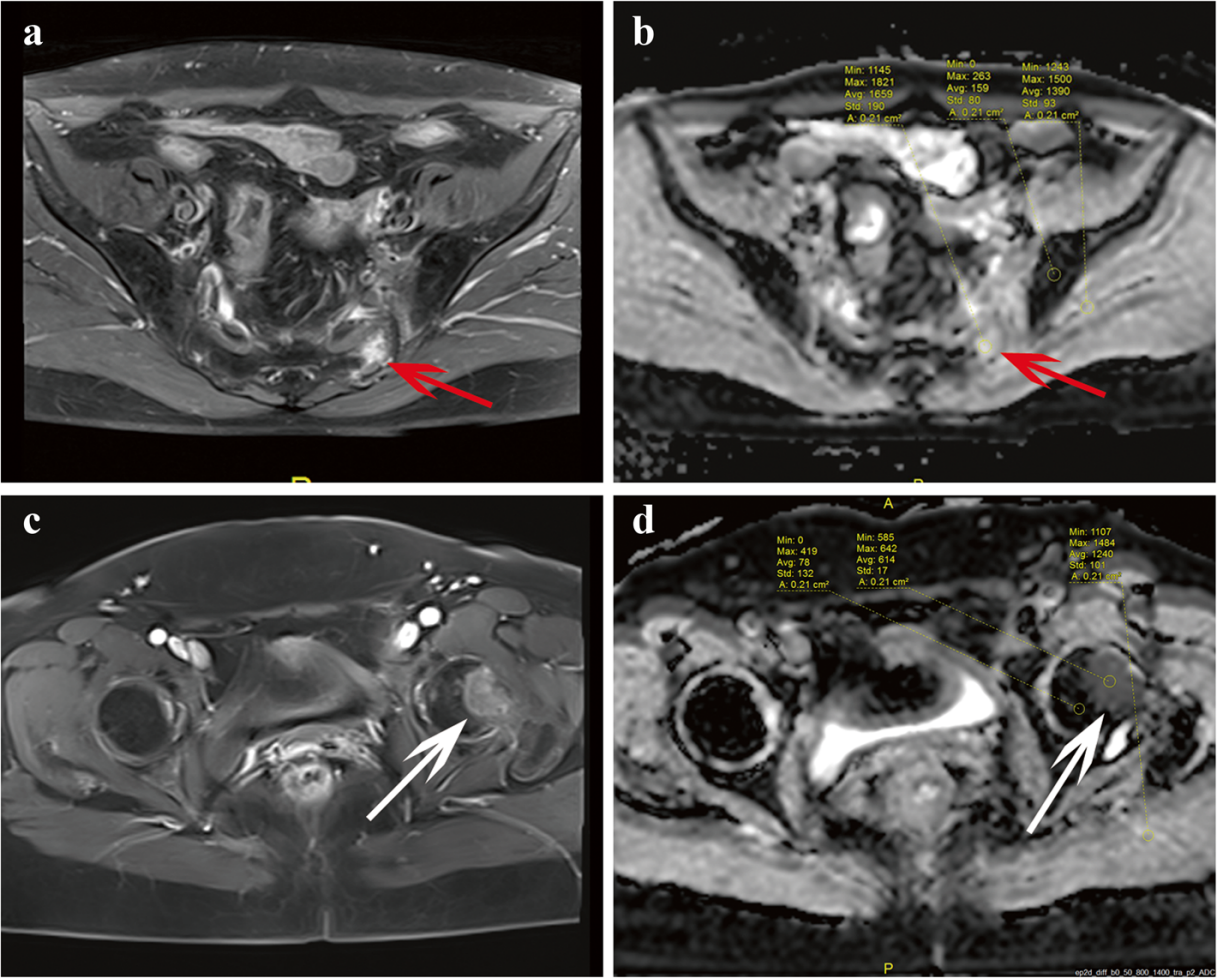
Schematic diagram of ADC parameters measurement. **a** ~ **b** ADC parameters measurement for occult IF; **a** An occult IF in the left sacrum (red arrow), which showed contrast enhancement on axial T1-weighted image post-contrast; **b** ADC parameters were measured on ADC map. **c** ~ **d** ADC parameters measurement for bone metastasis; **c** Bone metastasis in left femoral head (white arrow), which showed contrast enhancement on axial T1-weighted image post-contrast; **d** ADC parameters were measured on ADC map. The detailed approaches of ADC values measurement were as follows: at first ADC values of the lesion were measured manually drawing a region of interest (ROI) within contrast enhanced solid portion; the ADC_mean_, ADC_min_, ADC_max_ and ADC_std_ were documented, respectively. Then, the ROI was copied to adjacent normal bone marrow and muscle, and documented the mean ADC, respectively

The ADC measurement schematic diagram was shown in Fig. 2. Firstly, ADC value of lesions was measured manually drawing a region of interest (ROI) on the ADC map within an enhanced solid portion [13, 19]. For IFs without enhancement, the ROI was placed on bone marrow edema area [19]. The areas of necrosis (hyperintensity as high as fluid on T2-weighted images and without enhancement on contrast-enhanced T1-weighted images), calcification (hypointensity on both T2-weighted and T1-weighted images, without enhancement on contrast-enhanced T1-weighted images), or hemorrhage (hypointensity on T1-weighted images, hypo- or hyperintensity on T2-weighted images) were carefully avoided, and the mean ADC (ADC_mean_), minimum ADC (ADC_min_), maximum ADC (ADC_max_) and ADC standard deviation (ADC_std_) were documented for each lesion. Then, the ROI was copied to adjacent normal bone marrow and muscle, and the mean ADC was documented, respectively. Finally, the mean ADC ratio of lesion to normal bone [ADC_mean_ ratio (lesion/normal bone)] and the mean ADC ratio of lesion to [ADC_mean_ ratio (lesion/ muscle)] was determined, respectively. The inter-observer reproducibility of ADC measurement was assessed using interclass correlation coefficients (ICC). An ICC value > 0.75 indicates a good agreement on the measurement.

### Nomogram model development

Univariate analysis was performed to assess the statistic difference for each ADC parameter. Multivariate logistic regression analysis was performed to determine the independent ADC parameters in the differentiation of IFs from bone metastasis. Backward stepwise selection was used as the stopping rule based on the likelihood ratio test with Akaike’s information criterion [21]. A nomogram was constructed from the independent ADC parameters, and the calibration curve was used to evaluate the goodness-of-fit of the nomogram [22].

### Statistical analysis

R statistical software (version 3.3.1http://www.rproject.org/) was used for nomogram construction and calibration plots. Nomogram construction and calibration plots used the “rms” package. Other statistical analysis used the SPSS 16.0 (SPSS Inc., Chicago, IL, USA). The ADC parameters were expressed as median and interquartile range, and the univariate analysis for the ADC parameters used Mann–Whitney U test, the multivariate analysis used multivariate logistic regression analysis. The diagnostic performance of ADC parameters and the nomogram model for discrimination of PIFs and metastasis was determined by ROC analysis. *p* < 0.05 indicated a statistical significance.

## Results

### Patient characteristics

Of the 47 patients included, 59.6% (28/47) of patients were diagnosed with occult IFs and 40.4% (19/47) of patients were diagnosed with bone metastases. The median age was 64 years old for occult IF patients and 53 years old for bone metastasis patients. The median RT dose was 67 Gy for occult IF patients and 72 Gy for bone metastasis patients, respectively. The median interval time from RT to lesion detection by MRI was 16 months for IF patients and 24 months for bone metastases patients, respectively. Detailed patient characteristics are shown in Table 1**.**

**Table 1:** Patient characteristics

Characteristics	Occult IF (***n*** = 28)	Bone metastasis (***n*** = 19)
Median age (rang)	64 (41–81) years	53 (34–78) years
Menopausal status		
Postmenopausal	22	11
Premenopausal	5	8
Stage (FIGO)		
IA	1	0
IB	4	3
IIA	5	3
IIB	7	4
IIIA	6	2
IIIB	3	4
IV	2	3
Histopathology		
Squamous cell carcinoma	24	16
Adenocarcinoma	3	2
Adenosquamous cell carcinoma	2	1
Involvement multiple lesions	16	6
Median interval from RT to MRI (range)	16 (4–63) months	24 (8–54) months
Chemotherapy		
Yes	23	16
No	5	3
Median dose (rang)	67 (48–117) Gy	72 (56–124) Gy

*FIGO* International Federation of Gynecology and Obstetrics, *RT* radiotherapy

### Locations of occult PIFs and bone metastases

**Fig. 3:**
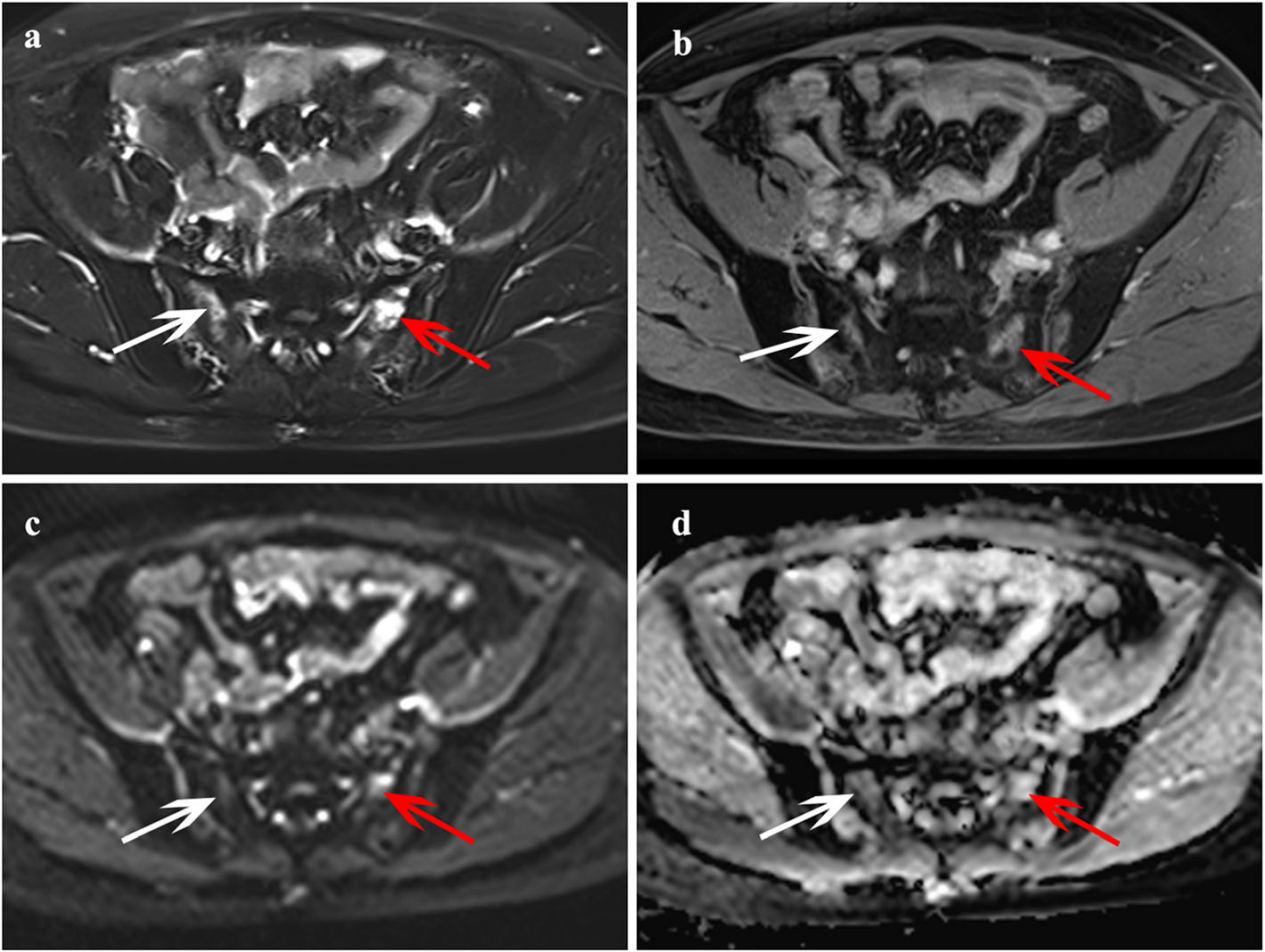
A 52-year-old female with cervical cancer after radiotherapy that was diagnosed with occult IFs (no fracture line detected on all images) in the bilateral sacrum. Axial FS T2-weighted image (**a**) showed hyperintensity in the bilateral sacrum (red arrow, white arrow, respectively), the lesions revealed contrast enhancement on axial T1-weighted image post-contrast (**b**). The lesions showed equisignal or slightly high signal intensity on DWI (**c**), and hyperintensity on ADC map (**d**)

**Fig. 4:**
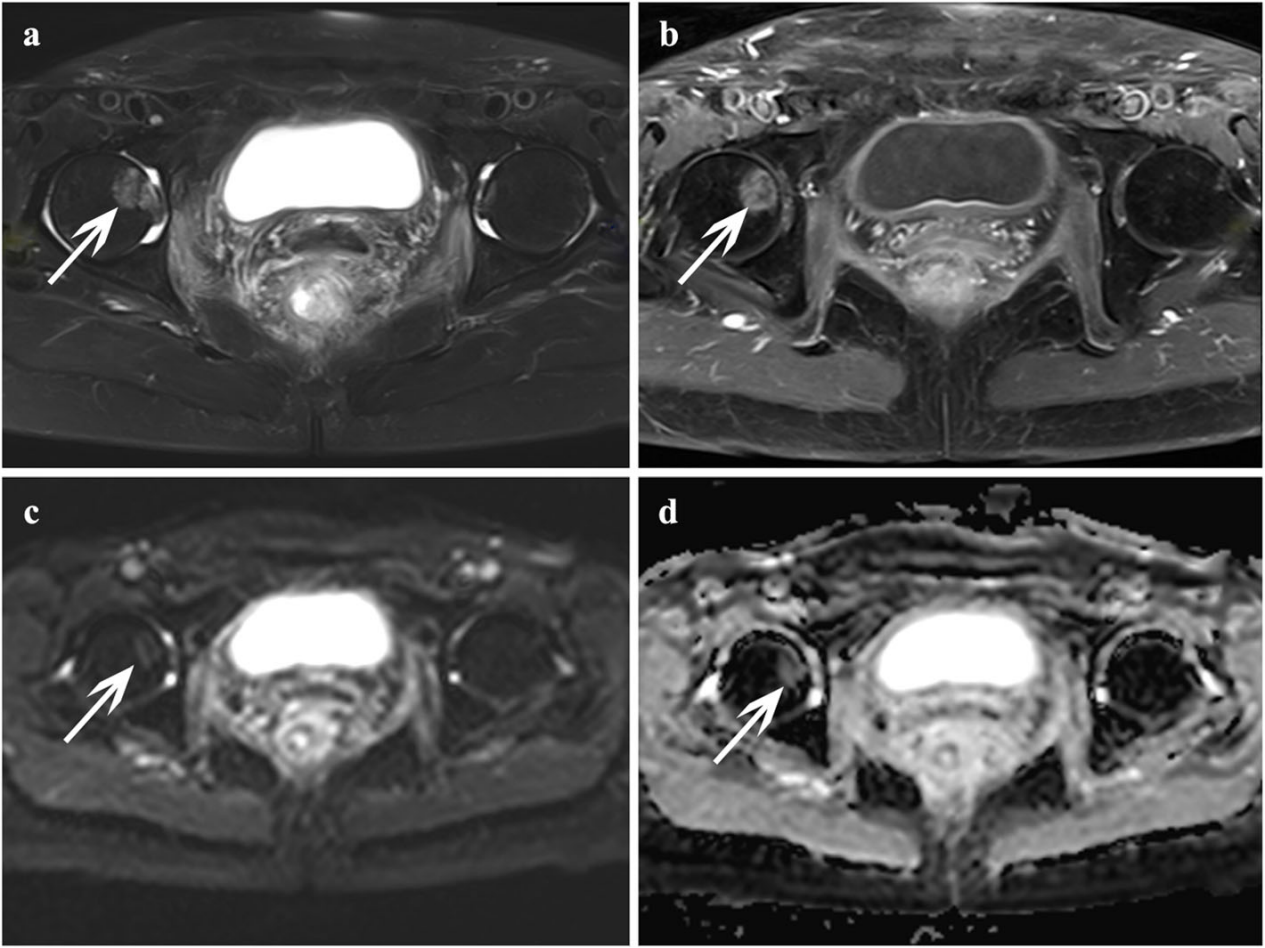
A 66-year-old female with cervical cancer after radiotherapy that was diagnosed with occult IFs (no fracture line detected on all images) of the right femoral head. **a** Axial FS T2-weighted image showed heterogeneous hyperintensity in the right femoral head (white arrow); **b** Contrast-enhanced T1-weighted image revealed marked and heterogeneous enhancement change. **c** The lesion showed equisignal or slightly high signal intensity on DWI, and hyperintensity on ADC map

**Fig. 5:**
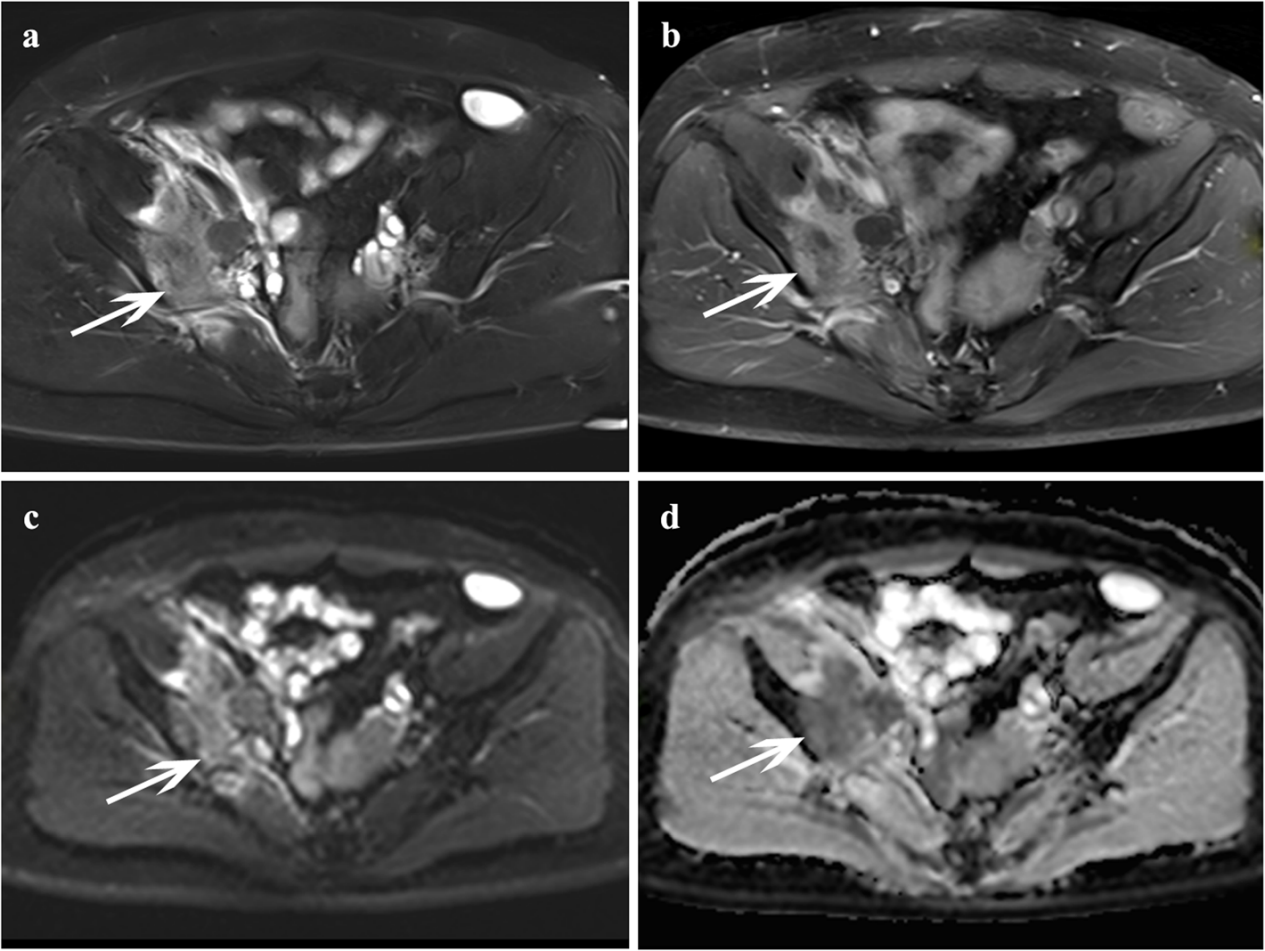
A 44-year-old female with cervical cancer after radiotherapy that was diagnosed with bone metastasis in the right ilium (white arrow). Axial FS T2-weighted image (**a**) showed a moderate hyperintensity solid mass in right ilium; the lesion showed contrast enhancement on axial T1-weighted image post-contrast, hyperintensity on DWI (**c**), and hypointensity on ADC map (**d**)

Of these 28 patients with occult IFs, 16 patients had multiple fractures and 12 patients had single fracture. A total of 48 IF lesions were identified based on the reference standard, occult IFs most commonly occurred in the sacrum (Fig. 3), accounting for 58.3% (28/48) of lesions; other locations included iliac wing (6 sites), femoral head (5 sites) **(**Fig. 4**)**, acetabulum (4 sites), pubic symphysis (2 sites), ischium (2 sites), and femoral neck (1 site). Of these 19 patients with bone metastases, 6 patients had multiple lesions and 12 patients had single lesion, and a total of 27 lesions were identified. Bone metastases most commonly occurred in the iliac wing (Fig. 5), accounting for 44.4% (12/27) of lesions; other locations included ischium (4 sites), sacrum (3 sites), femoral head (2 sites), acetabulum (2 sites), pubic symphysis (2 sites), and L5 centrum (2 sites). The number and location of lesions are shown in Table 2.

**Table 2:** Location and number of the all bone lesions

Locations	Occult IF (***n*** = 48)	Bone metastasis (***n*** = 27)
Sacrum	28 (58.3%)	3 (11.1%)
Acetabulum	4 (8.3%)	2 (7.4%)
Pubic symphysis	2 (4.2%)	2 (7.4%)
Femoral head	5 (10.4%)	2 (7.4%)
Ischium	2 (4.2%)	4 (14.8%)
Iliac wing	6 (12.5%)	12 (44.4%)
L5	0 (0%)	2 (7.4%)
Femoral neck	1 (2.1%)	0 (0%)

### Performance of univariate quantitative ADC parameters

As showed in Table 3, there were significant differences in the six ADC parameters between occult IFs and bone metastases (*p* < 0.01), and the inter-observer reproducibility of ADC parameters measurement indicated very good with ICC values, ranged from 0.787 to 0.933. Regarding ROC analysis, as showed in Table 4, the AUC values ranged from 0.743 to 0.887 in the differentiation of occult IFs from bone metastases; the ADC ratio (lesion/ muscle) showed an optimal diagnostic efficacy with a cut off value of 0.77, the sensitivity of 95.8% (46/48), the specificity of 81.5% (22/27), PPV of 90.2% (46/51), and NPV of 91.7% (22/24), respectively.

**Table 3:** Difference of ADC parameters between occult IF and bone metastasis group

ADC parameters	IF	Bone metastasis	***P*** value	ICC
	Median (Interquartile Range)	Median (Interquartile Range)		
ADC _mean_ (×10^−6^ mm^2^/s)	1336 (1097, 1519)	662 (495, 885)	< 0.001	0.933
ADC _min_ (×10^− 6^ mm^2^/s)	996 (734, 1228)	574 (429,764)	< 0.001	0.880
ADC _max_ (×10^−6^ mm^2^/s)	1527 (1228, 1828)	812 (571,1042)	< 0.001	0.826
ADC _std_ (×10^−6^ mm^2^/s)	132 (74, 200)	47 (32, 71)	< 0.001	0.907
ADC _ratio_ (Lesion/normal bone)	5.5 (4.1, 7.3)	2.8 (2.2, 3.4)	< 0.001	0.787
ADC _ratio_ (Lesion/lateral muscle)	1.2 (1.0, 1.3)	0.6 (0.5, 0.7)	< 0.001	0.840

*ADC* apparent diffusion coefficient, *ICC* intraclass correlation coefficient

**Table 4:** Performance evaluation of ADC parameters and nomogram model for differentiating occult IF and bone metastasis

Parameters	Cut off value	AUC (95% CI)	Sensitivity	Specificity	PPV	NPV
ADC _mean_ (×10^−6^ mm^2^/s)	990	0.853 (0.756,0.950)	85.4% (41/48)	85.2% (23/27)	91.1% (41/45)	76.7% (23/30)
ADC _min_ (×10^−6^ mm^2^/s)	828	0.743 (0.625, 0.861)	70.8% (34/48)	77.8% (21/27)	85.0% (34/40)	60.0% (21/35)
ADC _max_ (×10^−6^ mm^2^/s)	1044	0.837 (0.732, 0.942)	89.6% (43/48)	77.8% (21/27)	87.8% (43/49)	80.8% (21/26)
ADC _std_ (×10^−6^ mm^2^/s)	99.5	0.778 (0.670, 0.886)	66.7% (32/48)	88.9% (24/27)	91.4% (32/35)	60.0% (24/40)
ADC _ratio_ (Lesion/normal bone)	3.75	0.853 (0.756, 0.950)	85.4% (41/48)	85.1% (23/27)	91.1% (41/45)	76.7% (23/30)
ADC _ratio_ (Lesion/lateral muscle)	0.77	0.887 (0.793, 0.980)	95.8% (46/48)	81.5% (22/27)	90.2% (46/51)	91.7% (22/24)
Nomogram model (score)	0.97	0.921 (0.848, 0.995)	91.7% (44/48)	96.3% (26/27)	97.8% (44/45)	86.7%(26/30)

*AUC* area under the ROC curve, *PPV* positive predictive value, *NPV* negative predictive value

### Performance of combination multi-parametric ADC parameters

**Fig. 6:**
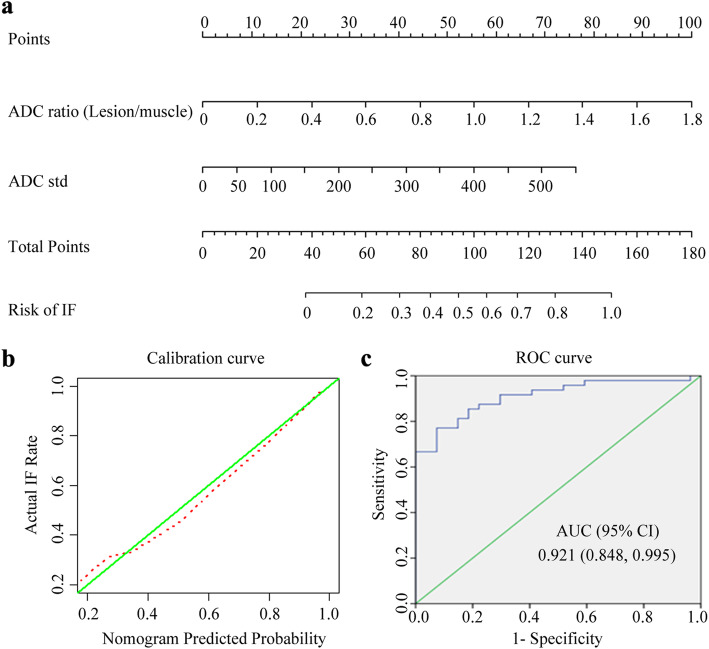
Development, calibration and performance evaluation of ADC-based nomogram for differentiating occult IF from bone metastasis. **a** Radiomics nomogram was developed from the ADC_std_ and ADC_mean_ ratio (lesion/muscle). **b** Calibration curves of the nomogram, calibration curves depict the calibration of the nomogram in terms of agreement between the predicted risk of ORN and observed outcomes. The 45^0^ green lines represent a perfect prediction, and the dotted red lines represent the predictive performance of the nomogram. The closer the dotted red line fit is to the green line, the better the predictive accuracy of the nomogram is. **c** ROC analysis of the nomogram model, the nomogram model showed a very good diagnostic performance with an AUC value of 0.921 (95%CI: 0.848, 0.995)

For multivariate analysis, ADC_std_ and ADC_mean_ ratio (lesion/muscle) were identified as independent factors in the differentiation of occult IFs from bone metastases. The odds ratio was 8.269 (95% confidence interval [CI]: 0.862, 79.287, 21.981) for ADC_std_ and 103.020 (95% CI: 9.387, 1130.500) for ADC_mean_ ratio (lesion/muscle), respectively. A diagnosis score was calculated for each lesion using a formula resulted from the two ADC parameters which were weighted by their coefficients: diagnosis score = − 8.8402 + (21,200 × ADC_std_) + [8.5044 × ADC_mean_ ratio (lesion/muscle)]. An ADC-based nomogram was constructed from the two independent factors; The nomogram (Fig. 6a**)** and calibration curve of the nomogram (Fig. 6b) were plotted. Based on the diagnosis scores for lesions, the performance of nomogram model was calculated using ROC analysis (Fig. 6c). As showed in Table 4, the nomogram model demonstrated very good diagnostic efficacy with an AUC value of 0.921 (95%CI: 0.848, 0.995), the sensitivity of 91.7% (44/48), the specificity of 96.3% (26/27), PPV of 97.8% (44/45), and NPV of 86.7% (26/30), respectively.

## Discussion

In this study, we preliminarily established a nomogram based on multiparameter apparent diffusion coefficients (ADC) to differentiate occult insufficiency fracture (IF) from bone metastasis after radiotherapy (RT) in cervical cancer. We found that multiple ADC parameters showed significantly statistical differences between occult IFs and bone metastases. ADC_std_ and ADC_mean_ ratio (lesion/muscle) were identified as independent factors to predict nomogram model. This ADC-based nomogram showed a greater performance in the discrimination of occult IF from bone metastasis, with an AUC of 0.921, the sensitivity of 91.7%, and the specificity of 96.3%, respectively. Thus, our results demonstrated that ADC-based nomogram might be used as a noninvasive, reliable and visual tool for detecting RT-reduced occult IFs.

As a specific type of stress fractures, IF most frequently occurs in the pelvis, which is caused by physiologic stress placed on lessened mineralization and elastic resistance bone [23, 24]. Pelvic radiotherapy has been considered as one of the most important risk factors in the development of IFs. Although IF is rarely life-threatening, it needs special attention as regards patients’ survival quality [25]. However, these RT-induced IFs are not well recognized in clinical practice, and IFs are usually occult and a considerable number of IFs may be misdiagnosed as bone metastases [26–29].

Recently, MRI is recommended as a sensitive and useful technique to detect IF, which has showed superior diagnostic efficacy compared with bone scan or CT [9–11]. Bone marrow edema with fracture line on MRI is a rather specific sign for diagnosing IFs [5, 11, 29]. However, MRI findings of IF may not always show a fracture line, and visualization of fracture line on MRI may partly relate to observer’s subjective experience. As showed in previous studies, the fracture line detection rate was ranged from 50 to 87% [9, 19, 20]. With the nonvisualization of fracture line, establishing the diagnosis of IF by conventional MRI alone is challenging, and these occult IFs may frequently be misinterpreted as metastasis.

Diffusion-weighted (DW) imaging has been widely used in the assessment of.

musculoskeletal disorders, including the discrimination of acute benign and malignant vertebral compression fractures (VCFs). Several studies indicated that DWI was a reliable diagnostic tool for distinguishing benign and malignant vertebral compression fractures [13, 14, 16, 17]. Regarding the discrimination of IF from bone metastasis, our previous study showed that the qualitative DWI provided additional value to the conventional MRI alone. However, we found that the improvement of diagnostic efficacy was varied based on observers’ experience [19]. In this study, we assessed the value of multiparameter ADCs for differentiating occult IF from bone metastasis after RT, the results demonstrated that the inter-observer reproducibility of ADC measurement between observers was very good, with an ICC ranged from 0.787 to 0.933. In line with previous studies using mean ADC in the discrimination of VCFs [16, 17], we found that ADC_mean_ was helpful for differentiating occult IF from bone metastasis, with an AUC of 0.853. In addition, other five ADC parameters were measured, including ADC_mean_ ratio (lesion/normal bone, lesion/muscle). Of the six ADC parameters, ADC _mean_ ratio (lesion/muscle) displayed the optimal discrimination performance with an AUC of 0.887. This was similar to a previous study in which ADC _mean_ ratio (lesion/muscle) showed great performance in the identification of benign and malignant VCFs [13].

Nomogram is a novel and helpful tool which has been applied in clinical research, including the differentiation of benign and malignant tumors [30], the prediction of tumor metastasis [31, 32], and the assessment of prognosis [33, 34]. Compared with previous studies in which only mean ADC value was assessed [18, 19], in order to seek a more useful and visual diagnostic tool to distinguish occult IF from bone metastasis, we successfully developed a nomogram model based on multiparameter ADC values to diagnose the RT-induced bone complication. Compared with individual ADC parameter, the ADC-based nomogram yielded a preferable diagnostic efficacy with an AUC value up to 0.921, especially improved the specificity to 96.3% and positive predictive value to 97.8%, respectively. Moreover, we found that this ADC-based nomogram demonstrated a very good calibration. Thus, the ADC-based nomogram might be used as a quantitative, reliable and visual tool in the differentiation of occult IFs from bone metastasis after RT. While other DWI techniques, including whole-body diffusion-weighted imaging (DWIBS), diffusion tensor imaging (DTI) and intravoxel incoherent motion Diffusion-Weighted MRI (IVIM-DWI) have been applied in the characterization of benign and malignant bone diseases [35–38], the value of these DWI techniques for diagnosing IF needs further investigation.

There were several limitations in our study. First, this was a retrospective study that preformed at single-institution; validation of the ADC-based nomogram had not been performed due to the relatively small sample size. Second, lack of histopathology confirmation as a gold standard. However, biopsy of the bone lesion for diagnosing IF may be generally impractical and at high risk, thus the possibility of selection bias in this study may not be thoroughly excluded. Third, as a retrospective nature, only b value of 800 was applied, value of other b values in the characterization of IF and bone metastasis is needed in further study.

## Conclusion

Several quantitative ADC parameters were helpful for identifying occult IFs and bone metastases after RT in cervical cancer. An ADC-based nomogram demonstrated a better performance than individual ADC parameter alone, particularly improved the diagnostic specificity and positive predictive value. ADC-based nomogram might be used as a reliable and visual tool in the differentiation of occult IFs from bone metastasis.

## Data Availability

The datasets used and/or analyzed during the current study available from the corresponding author on reasonable request.

## Abbreviations

IF: Insufficiency fracture
MRI: Magnetic Resonance Imaging
DWI: Diffusion-Weighted MR Imaging
ADC: Apparent diffusion coefficient
PPV: Positive predictive value
NPV: Negative predictive value
ROC: Receiver operating characteristic curve
AUC: Area under the receiver operating characteristic curve
RT: Radiotherapy
ICC: Intraclass correlation coefficient
